# Fate and Effect of Intravenously Infused Mesenchymal Stem Cells in a Mouse Model of Hepatic Ischemia Reperfusion Injury and Resection

**DOI:** 10.1155/2016/5761487

**Published:** 2016-02-11

**Authors:** T. C. Saat, S. van den Engel, W. Bijman-Lachger, S. S. Korevaar, M. J. Hoogduijn, J. N. M. IJzermans, R. W. F. de Bruin

**Affiliations:** ^1^Department of Surgery, Erasmus University Medical Center, Gravendijkwal 230, 3015 CE Rotterdam, Netherlands; ^2^Nephrology and Transplantation, Department of Internal Medicine, Erasmus University Medical Center, Gravendijkwal 230, 3015 CE Rotterdam, Netherlands

## Abstract

Liver ischemia reperfusion injury (IRI) is inevitable during transplantation and resection and is characterized by hepatocellular injury. Therapeutic strategies to reduce IRI and accelerate regeneration could offer major benefits. Mesenchymal stem cells (MSC) are reported to have anti-inflammatory and regeneration promoting properties. We investigated the effect of MSC in a model of combined IRI and partial resection in the mouse. Hepatic IRI was induced by occlusion of 70% of the blood flow during 60 minutes, followed by 30% hepatectomy. 2 × 10^5^ MSC or PBS were infused 2 hours before or 1 hour after IRI. Six, 48, and 120 hours postoperatively mice were sacrificed. Liver damage was evaluated by liver enzymes, histology, and inflammatory markers. Regeneration was determined by liver/body weight ratio, proliferating hepatocytes, and TGF-*β* levels. Fate of MSC was visualized with 3D cryoimaging. Infusion of 2 × 10^5^ MSC 2 hours before or 1 hour after IRI and resection showed no beneficial effects. Tracking revealed that MSC were trapped in the lungs and did not migrate to the site of injury and many cells had already disappeared 2 hours after infusion. Based on these findings we conclude that intravenously infused MSC disappear rapidly and were unable to induce beneficial effects in a clinically relevant model of IRI and resection.

## 1. Introduction

The liver has the unique ability of regeneration as a response to liver injury. In healthy individuals the liver can compensate an acute loss of 70% and return to its original mass within 30 days of resection [1]. Thanks to this remarkable feature and advances in surgical techniques, large (oncologic) liver resections as well as split and living donor liver transplantation are possible. However, in patients with chronic liver diseases, liver cirrhosis or malignancies, regeneration of the liver is often compromised due to chemotherapy [2], poor nutritional status [3], and the increasing age of patients [4]. Besides that, leaving a smaller fraction of residual liver after resection is a risk factor for postoperative morbidity due to hepatic dysfunction and infectious complications [5].

Liver transplantation (LTx) is the only life-saving treatment for end-stage hepatic diseases [6] and a treatment for patients with a primary liver tumor or colorectal metastases [7–9]. However, ischemia reperfusion injury (IRI), caused by interruptions of the hepatic blood flow, is inevitable during LTx and liver resection. Ischemia is characterized by ATP depletion and activation of anaerobic metabolic pathways, whereas reperfusion activates a cascade of pathways that causes further cellular damage and inflammation. IRI leads to a decreased regenerative capacity of hepatocytes, tissue necrosis, and apoptosis [10, 11]. Liver IRI is the leading cause of hepatocellular injury causing morbidity and mortality after LTx and may negatively affect liver regeneration after both postmortal and living donor LTx [12–14]. Taken together, potential therapeutic strategies to reduce hepatic IRI and accelerate liver regeneration could offer major benefits in both liver transplantation and resection.

Mesenchymal stem cells (MSC) are able to differentiate into different cell types, secrete growth factors, and have immunomodulatory and anti-inflammatory properties [15, 16]. Therefore, MSC are considered as a potential therapy to prevent or ameliorate hepatic IRI and stimulate liver regeneration.

In rodents, MSCs have the potential to reduce hepatic IRI by suppressing oxidative stress and inhibiting apoptosis [13, 17, 18]. Moreover, MSC improved liver regeneration in a resection model [13, 19, 20]. However, only few studies investigated the effect of MSC in a combined hepatic IRI and partial hepatectomy model [19, 21–23], while this model is relevant for translation to the clinical setting. Results from these studies suggest that MSC have beneficial effects on both IRI and regeneration. However, the use of MSC in a large animal model showed inconsistent results on IRI [24, 25]. Therefore, it remains unclear if and how MSC are able to prevent IRI and/or stimulate regeneration. The purpose of the present study is to investigate in a clinically relevant mouse model whether MSC are able to reduce hepatic IRI and stimulate liver regeneration after induction of hepatic IRI and partial liver resection.

## 2. Material and Methods

### 2.1. Animals

Male C57BL/6 (age 10–12 weeks, ~25 g) were obtained from Harlan (Horst, the Netherlands). Animals were kept under standard laboratory conditions and housed in individually ventilated cages (*n* = 3 animals/cage). The animals had free access to food and water (acidified with HCl). All experiments were performed with the approval of the institutional animal welfare committee (Protocol EMC2271).

### 2.2. Mesenchymal Stem Cell Cultures

MSC were isolated from abdominal adipose tissue of C57BL/6 mice. Syngeneic mouse MSC were used to avoid xenogeneic and allogeneic responses. The tissue was mechanically disrupted and enzymatically digested with 0.5 mg/mL collagenase type IV (Life Technologies, Paisley, UK) in RPMI 1640 Medium with glutaMAX (Life Technologies) for 30 min at 37°C under continuous shaking. The obtained cell suspension was washed and plated in tissue culture flasks in MEM-*α* with 15% fetal calf serum and 100 U/mL penicillin and 100 mg/mL streptomycin (1% p/s) (all Invitrogen, Germany). Nonadherent cells were removed after 2-3 days and subsequently culture medium was refreshed once a week. Plastic adherent cells were removed by trypsinization after reaching 70–80% confluency. Cells were maintained at 37°C, 5% CO_2_, and 95% humidity. MSC of passages 3–5 were used for experiments. Before intravenous infusion of 2 × 10^5^ MSC in 200 *μ*L PBS, cells were filtered through a 40 *μ*m sieve. The phenotype of these MSC used in previous studies was confirmed as they expressed CD44 and Sca-1, were negative for CD11b and CD45, were capable of differentiating into osteoblasts and adipocytes, and had the ability to inhibit conA stimulated lymphocyte proliferation [15].

### 2.3. Surgical Model

Mice were anesthetized by isoflurane/O_2_ inhalation. To maintain their body temperature mice were placed on a heating pad. All surgeries were performed between 9:00 am and 1:00 pm. After a midline laparotomy, partial hepatic ischemia was induced by occlusion of the blood flow of the left lateral and median liver lobes with a nontraumatic microvascular clamp for 60 minutes. The median and lateral lobe (approximately 70% of the liver) showed significant discoloration. After clamp removal, restoration of blood flow in the ischemic liver lobes causes reperfusion injury. Directly after removing the clamp, a partial hepatectomy (PH) was performed by resection of the left lateral liver lobe (approximately one-third of the liver). The abdomen was closed in two layers using Safil 5-0 (B. Braun, Germany). Mortality associated with this amount of damage to the liver was not observed. After surgery all mice received 0.5 mL of phosphate-buffered saline subcutaneously and were placed under a heating lamp until they recovered from anesthesia. Directly after surgery, all mice had free access to food and water.

Our pilot study showed that administration of 1 × 10^5^ MSC or 3 × 10^5^ was ineffective or showed a trend towards increased injury, respectively (Figure S1, in the Supplementary Material available online at http://dx.doi.org/10.1155/2016/5761487). Therefore, we chose a cell number of 2 × 10^5^ MSC as a potentially effective number of cells. We chose a preischemic time point of administration (2 hours before) and a time point 1 hour after ischemia to determine the effect of administration before and after inducing injury. Animals were treated intravenously via the tail vein with 2 × 10^5^ MSC in 200 *μ*L PBS or with PBS alone as vehicle control. Seven mice per group were infused 2 hours before hepatic IRI and resection or 1 hour after removing the clamp. They were sacrificed 6 and 48 hours and 5 days after surgery. Liver and blood samples were collected for further research. Control mice were sacrificed without undergoing any injection or intervention; their tissues were used as a baseline for mRNA expression levels.

### 2.4. Liver/Body Weight Ratio

Mice were weighed daily. The resected liver lobe was weighed after PH. At time of sacrifice, livers were removed, and animals and livers were weighed. The liver/body weight ratio was calculated as the wet liver weight divided by the total body weight of the mouse.

### 2.5. Hepatocellular Injury

Blood samples were collected at time of sacrifice 6 hours, 48 hours, or 5 days after surgery (*n* = 7 per time point). Sera were analyzed for alanine aminotransferase (ALAT) and aspartate aminotransferase (ASAT) levels at the Central Clinical Chemical Laboratory of the Erasmus University Medical Center.

Hemorrhagic necrosis was scored in 3 *μ*m thick paraffin liver sections stained with hematoxylin and eosin at a magnification of 100x by 2 independent observers blinded to the treatment (*n* = 5 per time point). Hemorrhagic necrosis was characterized by the loss of the cellular architecture and the presence of erythrocytes in necrotic areas. The percentage of hemorrhagic necrosis per microscopic field was scored with the following scoring system: 0%, 1% to 20% (≤20% necrosis per microscopic field), 21% to ≤40% necrosis per microscopic field, 41% to ≤60% necrosis per microscopic field, 61% to ≤80% necrosis per microscopic field, and 81% to 99% necrosis per microscopic field and 100% necrosis.

### 2.6. Immunohistochemistry

Frozen (5 *μ*m) liver sections from mice infused with PBS or MSC and sacrificed 6 hours, 48 hours, or 5 days after reperfusion and resection were stained with monoclonal antibodies against proliferating cell nuclear antigen (PCNA) (Abcam, UK). They were visualized with a horseradish peroxidase-conjugated secondary antibody (DAKO, Belgium). In 8 microscopic fields per section, the number of positive cells was counted by two observers (TS, SvED) blinded to the treatment at magnifications of 200 to 400x.

### 2.7. Quantitative RT-PCR

Total RNA was extracted from frozen liver tissue using Trizol reagent (Invitrogen, Breda, the Netherlands), purified by a DNase treatment (RQ1 RNase-Free DNase; Promega Benelux B.V., Leiden, the Netherlands), and reverse transcribed to complementary DNA using random hexameter primers and Superscript II RT (both from Invitrogen, Breda, the Netherlands) according to manufactures instructions. Quantitative real-time PCR was performed using a MyiQ single-color Real-Time PCR Detection System with SYBR Green incorporation (both from Bio-Rad Laboratories B.V., Veenendaal, the Netherlands; primer sequences are available upon request).

B2M and HPRT were used as housekeeping genes; IL-6 and TNF-*α* were used as genes indicative for inflammation. To assess cytoprotection we measured HO-1 and IL-10 as anti-inflammatory genes.

TGF-*β* was used as a marker for regeneration. Primers used to amplify the genes of interest were obtained from Sigma, the Netherlands. The relative expression was calculated as 2^−(ΔCt sample−ΔCt control)^, corrected for expression levels in healthy control animals. Mice without any infusion and surgery were used as healthy controls. Each sample was tested at least in duplicate.

### 2.8. MSC Tracking by 3D Cryoimaging

One batch of MSC was labeled with fluorescent Qtracker 605 beads according the manufacturer's description (stains viable MSC; Life Technologies, Grand Island, NY, USA). 2 × 10^5^ labeled MSC were infused in the tail vein directly after hepatic IRI. Two hours after the induction of IRI the mouse was sacrificed by isoflurane overdose and the whole mouse was snap frozen in Tissue-Tek O.C.T. Compound (Sakura Finetek Europe B.V., Alphen aan den Rijn, the Netherlands). 3D anatomical and molecular fluorescence videos were generated by CryoViz*™* (BioInVision, Mayfield Village, OH, USA), allowing the detection of single labeled cells.

### 2.9. Statistical Analysis

Data were expressed as mean ± standard error of the mean. Differences between groups were analyzed by Mann-Whitney *U* tests using SPSS (version 21). Differences were considered significant at *P* values less than 0.05.

## 3. Results

### 3.1. Effect of Mesenchymal Stem Cells on Liver Damage

**Figure 1:**
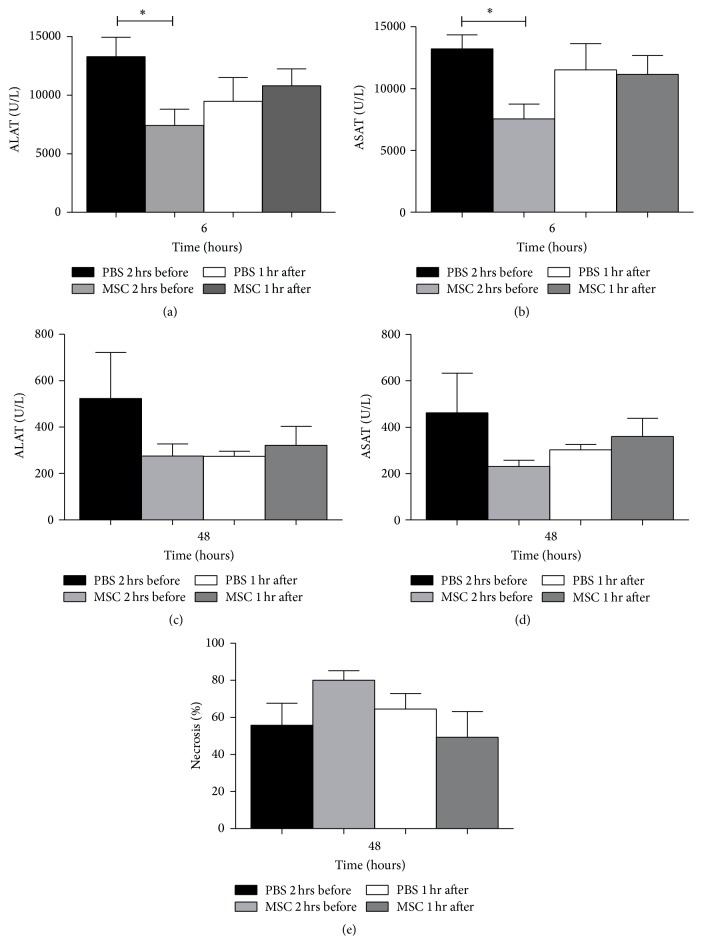
Hepatocellulair injury. (a) Six hours after IRI and PH, the serum ALAT concentration was significantly lower in mice treated with MSC 2 hours before hepatic IRI and PH compared to their PBS control. (b) Serum ASAT concentration, six hours after reperfusion, was significantly lower in mice treated with MSC 2 hours before IRI and PH compared to their PBS controls. (c) Forty-eight hours after IRI and PH there were no significant differences in serum ALAT levels between mice treated with MSC or PBS. (d) Serum ASAT concentrations showed no significant differences forty-eight hours after IRI and PH between mice infused with MSC or PBS. (e) Forty-eight hours after IRI and PH, livers from mice treated with MSC showed no differences in amount of necrosis compared to their PBS controls. The data are expressed as means ± SEM ((^*∗*^ *P* < 0.05) versus their PBS controls).

Liver damage was evaluated by measuring serum transaminases and histological damage. Six hours after reperfusion and partial hepatectomy (PH), serum ALAT (7400 ± 1392 U/L) and ASAT (7529 ± 1202 U/L) levels were significantly lower in mice infused with MSC 2 hours before IRI and PH compared with their PBS controls (ALAT 13271 ± 1644 U/L) (ASAT 13207 ± 1131 U/L) (*P* = 0.015, Figure 1(a)) (*P* = 0.005, Figure 1(b)). Mice infused with MSC 1 hour after reperfusion and PH showed no significant difference compared with their PBS control group (Figures 1(a) and 1(b)). 48 hours after reperfusion and PH, there were no significant differences in transaminases between mice infused with MSC 2 hours before or 1 hour after reperfusion and PH compared to the mice infused with PBS (Figures 1(c) and 1(d)).

Histological examination of livers 48 hours after reperfusion and PH (Figure 1(e)) revealed no significant differences in amount of hemorrhagic necrosis between mice treated with MSC compared to controls.

### 3.2. Effect of Mesenchymal Stem Cells on Inflammation

**Figure 2:**
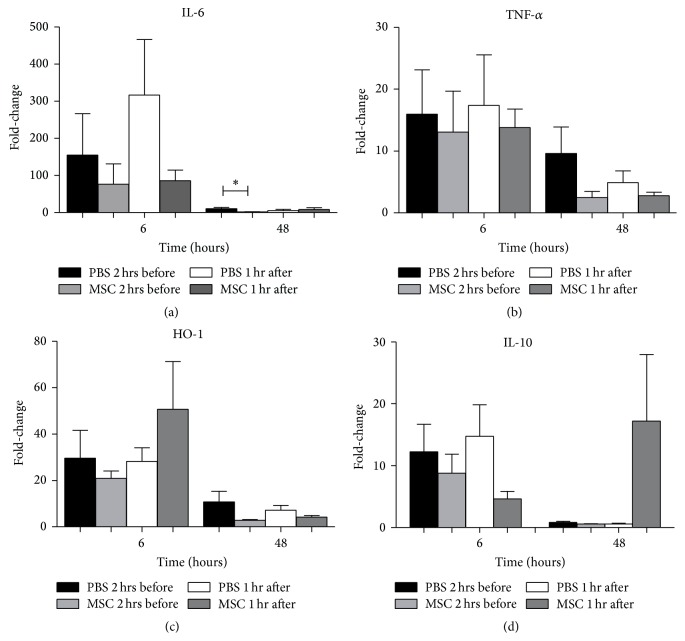
(Anti-)inflammatory and cytoprotective response. (a) mRNA levels of inflammatory marker IL-6 showed no differences six hours after reperfusion. Forty-eight hours after reperfusion mice treated with MSC 2 hours before IRI and PH showed significant lower expression levels of IL-6 compared to PBS treated mice. (b) Inflammatory markers TNF-*α* showed no significant difference after reperfusion. (c) Cytoprotective gene HO-1 showed no significant differences between mice treated with MSC or PBS at both time points. (d) Anti-inflammatory gene IL-10 showed no significant differences between MSC or PBS treated mice. The data are expressed as means ± SEM ((^*∗*^ *P* < 0.05) versus their PBS controls).

We investigated the effect of MSC on the inflammation induced by IRI by measuring the expression levels of IL-6 and TNF-*α* compared with the PBS treated controls. Six hours after reperfusion and PH, there were no significant differences in expression levels of IL-6 between both groups. However, 48 hours after reperfusion expression levels of IL-6 were significantly lower in mice treated with MSC 2 hours before surgery compared to their PBS controls (*P* = 0.013). Mice treated with MSC 1 hour after surgery showed no differences (Figure 2(a)). TNF-*α* did not show significant differences in expression levels after reperfusion and PH in mice infused with MSC or PBS (Figure 2(b)).

In response to hepatic IRI, the cytoprotective gene HO-1 is upregulated in an attempt to protect the liver against antioxidants. In previous studies, we found that upregulation of HO-1 was associated with reduced the liver damage [26]. In the present study, infusion of MSC had no effect on the expression of HO-1 compared to their PBS control group (Figure 2(c)). Anti-inflammatory gene IL-10 showed no differences between mice treated with MSC compared to their PBS controls after both time points (Figure 2(d)).

### 3.3. Effect of Mesenchymal Stem Cells on Liver Regeneration

**Figure 3:**
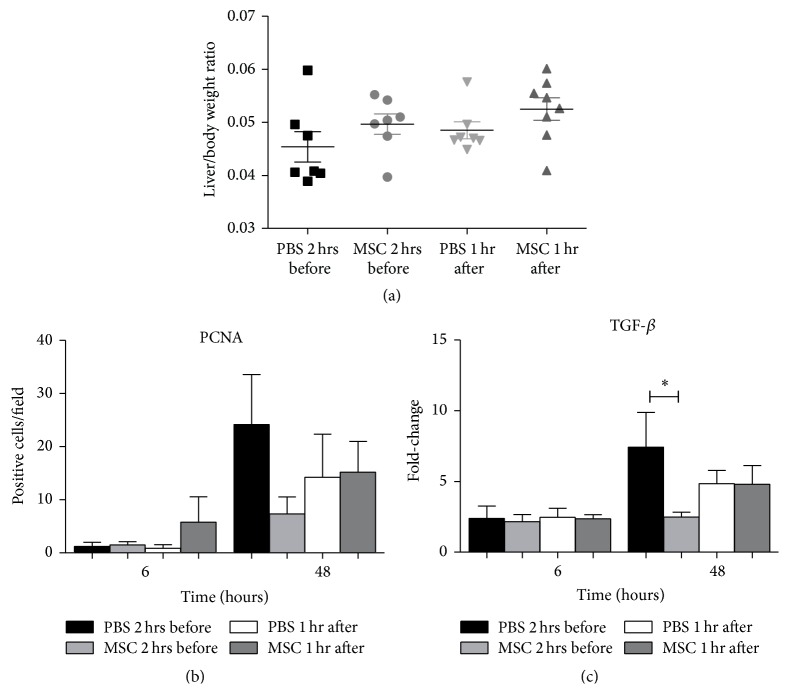
Liver regeneration. (a) Liver/body weight ratio 5 days after IRI and PH showed no significant differences between MSC or PBS treated mice. (b) After 6 and 48 hours after IRI and PH the numbers of PCNA positive cells were not significantly different. (c) Regeneration marker TGF-*β* showed significantly higher expression levels forty-eight hours after reperfusion in mice treated with PBS 2 hours before IRI and PH. The data are expressed as means ± SEM ((^*∗*^ *P* < 0.05) versus their PBS controls).

The remarkable capacity of the liver to regenerate is important for the postoperative liver function and is influenced by hepatic IRI. We determined liver/body weight ratios 5 days after hepatic IRI and PH of mice infused with MSC 2 hours before or 1 hour after hepatic IRI and PH, compared with their PBS controls. There were no significant differences in liver weight/total body weight ratios between mice treated with MSC or their PBS controls (Figure 3(a)).

Next we investigated hepatocyte proliferation with a PCNA staining (Figure 3(b)). Six hours after hepatic IRI and PH, there was no significant difference between mice infused with MSC or PBS. Forty-eight hours after IRI and PH, the PCNA index peaked but there were no significant differences between the MSC and the PBS groups.

Transforming growth factor beta (TGF-*β*) is a protein involved in the termination response of liver regeneration [27]. Six hours after IRI and PH, there were no differences between the MSC infused mice and their PBS controls. Forty-eight hours after IRI and PH expression levels of TGF-*β* were significantly lower in mice infused with MSC 2 hours before surgery compared to their PBS controls (*P* = 0.025); mice infused with MSC 1 hour after surgery showed no significant differences (Figure 3(c)).

### 3.4. Fate of Intravenously Infused Mesenchymal Stem Cells

**Figure 4:**
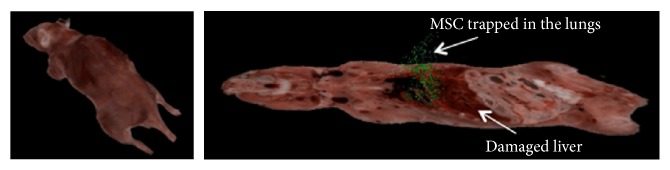
In vivo cryoimaging of labeled MSC. MSC labeled with Qtracker 605, which stains live cells and infused directly after and imaged 2 hours after hepatic IRI are present in the lungs and not in the damaged liver. Many of the liver MSC are trapped in the lungs, but the majority of infused cells cannot be detected, suggesting that these cells are dead.

Using CryoViz whole body imaging, MSC can be detected at single cell level and quantified. Luk et al. (unpublished data) showed by CryoViz imaging that intravenously infused MSC disappear for over 90% within 24 hours after infusion in a healthy mouse but also in a mouse with renal IRI and did not migrate to the damaged organ. We infused 2 × 10^5^ labeled MSC directly after the induction of hepatic IRI. Two hours after IRI the mouse was sacrificed and the MSC were traced. By far, most cells were found in the lungs (45584 cells); in the damaged liver 3693 cells were present. A few cells were found in other organs and 1218 cells were found in the rest of the mouse. These data show that MSC infused intravenously 2 hours before hepatic IRI are trapped in the lungs and do not migrate to the injured liver (Figure 4 and Table 1). Importantly, 2 hours after infusion of 2 × 10^5^ MSC, only 50.607 MSC were found, indicating that many of the infused cells disappeared within 2 hours.

## 4. Discussion

Combined IRI and inadequate tissue regeneration are major causes of morbidity and mortality after LTx and liver resection [12, 13, 28]. Animal models showed beneficial effects of MSC therapy on repair of injured organs and are able to ameliorate inflammatory processes [13, 29–31]. However, the role of MSC therapy in a clinically relevant model of combined hepatic IRI and partial liver resection remains to be established.

In the present study we demonstrated that infusion of 2 × 10^5^ MSC 2 hours before hepatic IRI and PH significantly decreased ASAT and ALAT levels compared to their PBS controls six hours after IRI and PH but did not decrease the amount of IRI induced hemorrhagic necrosis. In addition, MSC infused before or after hepatic IRI and PH showed no beneficial effect on liver damage or regeneration. Although the kinetics of markers of injury and inflammation after IRI and proliferation and termination after resection are relatively well known, their behavior following combined IRI and resection is less clear [32, 33]. Serum transaminase levels were significantly lower at 6 hours after IRI and PH in mice that were infused with MSC 2 hours before surgery. Intrahepatic IL-6 mRNA and TNF-*α* levels did not provide consistent results. Cytoprotective gene HO-1 showed a trend towards higher levels of expression in mice treated with MSC 1 hour after surgery, whereas IL-10 did not. Despite the significant differences in transaminases between MSC and PBS treated mice, the amount of liver necrosis at 48 hours after surgery, which can be considered the gold standard to measure hepatocellular injury, did not differ between PBS treated controls and any of the MSC treated groups. These data show that MSC infusion may have modulated the early inflammatory response but had no effect on IRI induced liver damage.

Liver regeneration as assessed by liver/body weight ratio was not significantly different between groups, although there was a trend towards a higher ratio in the MSC treated animals. However, this is not supported by numbers of proliferating cells and TGF-*β* expression at 48 hours after IRI. However, 48 hours after IRI and PH, TGF-*β* expression attained significant higher levels in the PBS infused mice before IRI and PH compared to MSC treated mice. On the one hand, this might be explained by the role of TGF-*β* as a key mediator of tissue fibrosis [34–36]. Rats treated with hepatocyte growth factor (HGF) expressing MSC after small-for-size liver transplantation showed a decrease in hepatic fibrosis compared to rats treated with PBS [37]. In this model, a suppressed production of TGF-*β* is correlated with decreased fibrosis, suggesting that the balance between TGF-*β* and HGF plays a critical role in the recovery or fibrogenesis of the injured tissues. On the other hand, higher levels of proliferation and TGF-*β* in control mice in our study may also suggest a more rapid regenerative response in the absence of MSC.

Our findings are in contradiction with the results of Saidi et al. [23]. Using a mouse model with 60 minutes of partial IRI with 70% hepatectomy, mice were treated with 1-2 million human adipose-derived MSC that were administered intravenously 30 minutes before ischemia. Mice treated with MSC showed improved survival, lower levels of ALAT and serum IL-6, and increased numbers of PCNA positive cells. Labelled MSC localised in the liver for 9 days.

How MSC exert their effect is still matter of debate. Using 3D cryoimaging, we were able to show that intravenously infused MSC are exclusively trapped in the lungs and do not migrate to the injured liver and that many infused cells have disappeared from the body 2 hours after infusion. These data support recent findings that infused MSC do not migrate to sites of injury but are trapped in microcapillary networks, have a short lifespan, and exert their action via paracrine mechanisms [15, 19, 38–41]. MSC have been observed in liver, spleen [23, 42, 43], and injured organs [26, 40, 42] after intravenous infusion. The viability of these cells is not well known, and it has been suggested that they may represent dead or phagocytosed MSC. Despite the hypothesis that the detected MSC are dead, a variety of animal models showed beneficial effects of MSC [13, 23, 28, 43, 44] lending further support to the notion that delivery of MSC to the injured organ is not required for their beneficial effects [16, 41].

In our study robust beneficial effects of MSC on liver injury and regeneration are lacking while in other studies they have been shown to be effective in ameliorating hepatic IRI and promote liver regeneration [20, 23, 27, 28, 43, 44]. The variable outcomes in animal studies have many reasons. The number of MSC is a critical factor, yet it differs between studies, with reported numbers between 1–3 million MSC in rats and 0.5–2 million MSC in mice [13, 22, 23, 38, 45]. We used 2 × 10^5^ MSC since higher numbers of cells induced liver damage (Figure S1). The route of administration, which can be local via the portal vein [13] or systemic via the tail vein [21], might play an important role in the outcome after MSC infusion. Since it is now known that MSC do not migrate beyond the lungs after intravenous infusion, the systemic route may not be the best route for MSC administration. MSC delivered via the portal vein were detected in the liver until postoperative day 7. However, the effect on liver damage and regeneration was comparable to infusion of MSC via the tail vein [13]. Also, the timing of MSC administration might play an important role in the outcome after IRI and PH.

## 5. Conclusion

Intravenous infusion of 2 × 10^5^ MSC 2 hours before or 1 hour after partial IRI and 30% PH did not ameliorate liver damage or improve regeneration. Using 3D cryoimaging, we showed that many of the infused MSC disappear within 2 hours and that the remaining cells are trapped in the lungs and do not migrate towards the damaged liver. More research is needed for the sources of these inconsistent results to improve the reliability of MSC therapy and get closer to translation to the clinical arena.

## Supplementary Material

Figure S1: Hepatocellular injury after infusion of PBS, 1x105 or 3x105 MSC directly after IRI and PH.

## Figures and Tables

**Table 1:** Number of detected MSC 2 hours after hepatic IRI.

Specimen	Injected after IRI	Liver	Lung	Spleen	Left kidney	Right kidney	Rest of mouse
2 hours after IRI	200 000	3694 (1.8%)	45 584 (22.8%)	64 (0.03%)	18 (0.009%)	29 (0.01%)	1 218 (0.6%)
