# Supplementary material for: Fate and Effect of Intravenously Infused Mesenchymal Stem Cells in a Mouse Model of Hepatic Ischemia Reperfusion Injury and Resection
Source: Stem Cells Int. 2016 Feb 11;2016:5761487. doi: 10.1155/2016/5761487 (PMC4766354; doi:10.1155/2016/5761487)
Supplement: Supplementary file 1 — Figure S1: Hepatocellular injury after infusion of PBS, 1x10^5^ or 3x10^5^ MSC directly after IRI and PH. [file 5761487.f1.pdf]

Figure S1

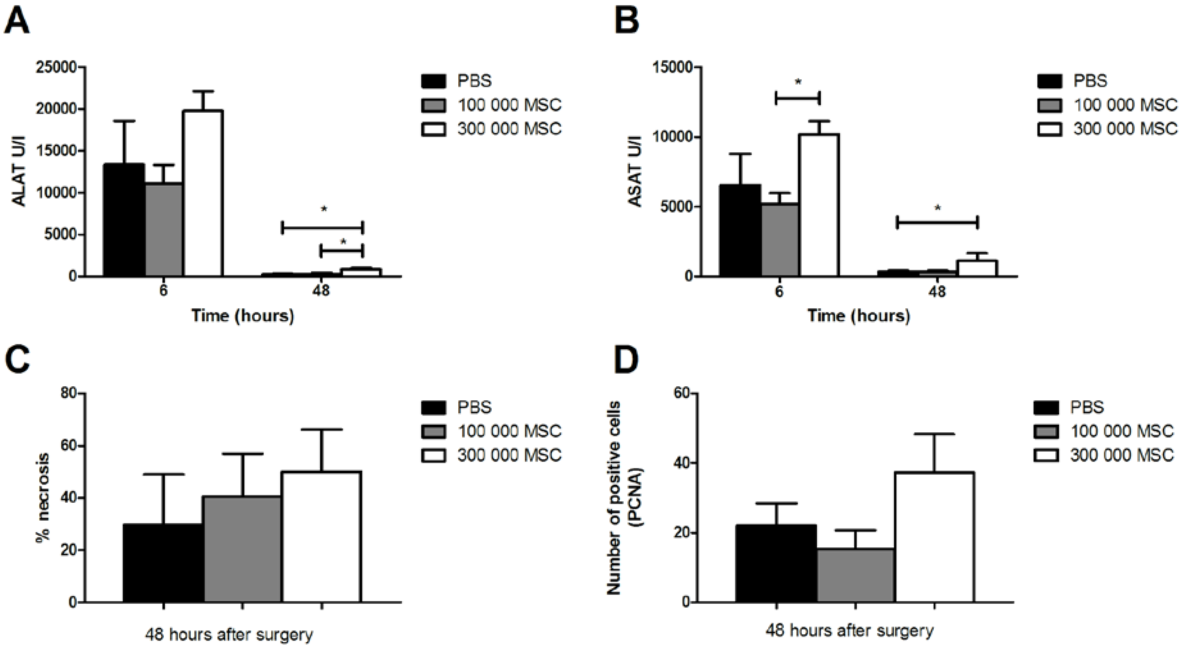

Figure S1: **Hepatocellular injury after infusion of PBS, 1x10^5 or 3x10^5 MSC directly after IRI and PH. (A**) Six hours after IRI and PH serum ALAT levels showed no significant differences between PBS, 1x10^5 or 3x10^5 MSC infused mice. Forty-eight hours after IRI and PH, mice infused with 3x10^5 MSC showed significant higher serum levels of ALAT compared to the PBS and 1x10^5 MSC treated mice. **(B)** Mice infused with 3x10^5 MSC showed significant higher serum ASAT levels six hours after IRI and PH compared with mice infused with 1x10^5 MSC. Forty-eight hours after IRI and PH mice treated with 3x10^5 MSC showed significant higher levels of ASAT compared to PBS treated mice. **(C)** Forty-eight hours after IRI and PH there was no difference in percentage of necrosis in liver tissue between mice infused with PBS, 1x10^5 or 3x10^5 MSC. However, mice infused with 3x10^5 MSC show a trend towards higher percentage of necrosis compared to PBS treated mice. **(D)** The number of PCNA positive cells was not significantly different. However, mice treated with 3x10^5 MSC might show a trend towards higher number of proliferating cells compared to PBS treated mice.

These data suggest that there is more liver damage in mice treated with 3x10^5 MSC, and hence a stronger regenerative response. The data are expressed as means ± SEM, ((*P<0.05) vs PBS or 1x10^5 MSC infused mice).
